# Medical Hoax

**Published:** 1841-05

**Authors:** 


### MEDICAL HOAX.

During the early part of last month, two or three of our younger Physicians made a few Galvanic experiments on a criminal recently hung in this city, by which some slight muscular contraction was excited, though not quite as much as usual in such cases.

On this state of fact a correspondent of one of our newspapers proceeded to construct a report which, as we have been told, for we did not read it, represented the criminal as raised to his feet and made to walk about the room, performing a variety of pantomimic gestures. We perceive by the newspapers, that this report has been regarded as authentic, and as it stated that another might be looked for in our Journal, we feel it a duty to make that which we have just penned.

We cannot but regret, that our newspaper press should so often give utterance to fabrications intended to deceive the public. What is a hoax but a falsehood? And what is there in the publication of a falsehood that can be approved? If our brethren of the political press cannot make their papers interesting to their readers, without imposing on them the creations of imagination as facts relating to passing events, they would do well to abandon the profession for some calling in which the price of bread is not the sacrifice of veracity, or the practice of imposture. D.

